# Bacterial isolates and drug susceptibility patterns of ear discharge from patients with ear infection at Gondar University Hospital, Northwest Ethiopia

**DOI:** 10.1186/1472-6815-13-10

**Published:** 2013-08-06

**Authors:** Dagnachew Muluye, Yitayih Wondimeneh, Getachew Ferede, Feleke Moges, Tesfaye Nega

**Affiliations:** 1 School of Biomedical and Laboratory Sciences, College of Medicine and Health Sciences, University of Gondar, P.O. Box 196, Gondar, Ethiopia; 2 Unit of Bacteriology, Gondar University Hospital, P.O. Box 196, Gondar, Ethiopia

**Keywords:** Ear infection, Bacterial isolates, Drug susceptibility, Gondar university hospital

## Abstract

**Background:**

Ear infection is a common problem for both children and adults especially in developing countries. However in Ethiopia particularly in the study area, there is no recent data that shows the magnitude of the problem. The aim of this study was to determine the bacterial isolates and their drug susceptibility patterns from patients who had ear infection.

**Method:**

A retrospective study was conducted from September, 2009 to August, 2012 at Gondar University Hospital, Northwest Ethiopia. Ear discharge samples were cultured on MacConkey agar, blood agar and chocolate agar plates. A standard biochemical procedure was used for full identification of bacterial isolates. Antimicrobial susceptibility tests were done on Mueller-Hinton agar by using disk diffusion method. Data were entered and analyzed by using SPSS version 20 software and P-value of < 0.05 was considered statistically significant.

**Result:**

A total of 228 ear discharge samples were tested for bacterial isolation and 204 (89.5%) cases were found to have bacterial isolates. From the total bacterial isolates, 115 (56.4%) were gram negative bacteria and the predominant isolate was *proteus species* (27.5%). Of individuals who had ear infection, 185 (90.7%) had single bacterial infection while 19 (9.3%) had mixed infections. Under five children were more affected by ear infection. The prevalence of ear infection was significantly high in males (63.7 vs 36.3%) (P = 0.017). Of all bacterial isolates, 192 (94.1%) had multiple antibiotic resistant pattern. Non Lactose Fermenter Gram Negative Rods (46.0%), *Klebsella species* (47.7%) and *Pseudomonas species* (48.5%) were resistant against the commonly used antibiotics.

**Conclusion:**

The prevalence of ear infection was very high in the study area. Majority of the bacterial isolates were resistant to multiple antibiotics. Hence antibiotics susceptibility test is mandatory before prescribing any antibiotics.

## Background

Ear infection is an inflammation of the ear and ear discharge is one of the commonest symptoms of ear infection [1]. About 65-330 million people suffer from ear infection worldwide and 60% of them had significant hearing impairment [2]. The health-economic burden of ear infection is also severe especially in Africa and other developing nations where the disease prevalence is estimated as high as 11% [3].

Ear infection is a common problem for both children and adults but the magnitude is different in different countries. Anatomically the children’s Eustachian tube is shorter, more horizontal with a more flaccid cartilage which can easily impair its opening and hence ear infection is a major health problem of them especially in those with poor socioeconomic status [4].

The etiologies and prevalence of ear infection is different indifferent geographical areas [5,6]. According to World Health Organization (WHO) survey, countries can be clustered into those having low ear infection when a prevalence rate of ear infection among children is between 1-2% and high when it is 3-6% and Ethiopia belongs to the latter category [7]. Though ear infection can be caused by viruses and fungi infections, the major causes of ear infection are bacterial isolates such as *Pseudomonas aeruginosa, Staphylococcus aureus*, *Proteus mirabilis, Klebsiella pneumonia* and *Escherichia coli* which are found in the skin of the external ear and enter into the middle ear through a chronic perforation [8,9].

In addition, antimicrobial resistance profile of bacteria varies among population because of the difference in geography, local antimicrobial prescribing practices and prevalence of resistant bacterial strains in a given area [10]. So there should be up to date information on microbial resistance pattern at national and local levels to guide the rational use of the existing antimicrobial drugs.

In Ethiopia particularly in the study area, there is no such type of recent data that shows the magnitude of the problem. Therefore, the aim of this study was to determine the bacterial isolates and their drug susceptibility patterns from patients who gave ear discharge samples at Gondar University Hospital.

## Methods

### Study design, area and period

A retrospective study was conducted from September, 2009 to August, 2012 at Gondar University Hospital, Northwest Ethiopia. This University Hospital provides inpatient and outpatient services for more than 5 million populations surrounding it.

### Study participants and data collection

The study participants were all individuals who had complain of ear infection and those who provide ear discharge sample at Gondar University Hospital during the study period. Socio-demographic and laboratory results which contain different bacterial isolates and drug susceptibility patterns of patients who had ear discharges were collected from the University Hospital Microbiology Laboratory unit registration books by using standard data collection format.

### Culture and identification

According to the standard operation procedures, the ear discharge samples were collected aseptically by using cotton swab techniques from different OPDs and wards of the University Hospital and transported to microbiology laboratory. Ear discharge samples were cultured on MacConkey agar, blood agar and chocolate agar plates and then incubated aerobically at 37°C for 24 hours. The swarming feature of proteus species were managed by sub culturing mixed colonies in to MacConkey agar that contains bile salt and by adding 90% ethanol. Pure isolates of bacterial pathogen were preliminary characterized by colony morphology, gram-stain and catalase test. Bacterial species were identified as per the standard microbiological methods [11].

### Antimicrobial susceptibility testing

Antimicrobial susceptibility tests were done on Mueller-Hinton agar (Oxoid, England) using disk diffusion method [12]. The antimicrobial agents tested were tetracycline (30 μg), penicilin G (10 μg), erythromycin (15 μg), chloramphenicol (30 μg), gentamicin (10 μg), ciprofloxacin (5 μg), norfloxacillin (10 μg), cotrimoxazole (25 μg), ceftriaxone (30 μg), ampicillin (10 μg) and amoxycillin (10 μg) (Oxoid, England). The drug susceptibility pattern was interpreted according to Clinical and Laboratory Standards Institute (CLSI, 2006) (formerly known as National Committee for Clinical Laboratory Standards/NCCLS) [13]. Reference strains of *E. coli* ATCC 25922 and S*. aureus* ATCC 25923 were used for quality control for antimicrobial susceptibility tests [13].

### Statistical analysis

Data were cleaned manually and entered and analyzed by using SPSS version 20 software. Chi-square test was employed to compare the proportion of bacterial isolates with patients’ demographic information and comparison of antimicrobial resistances. P-value < 0.05 was considered statistically significant.

### Ethical considerations

Ethical clearance was obtained from the Institutional Review Board of University of Gondar. A supportive letter was also obtained from College of Medicine and Health Sciences and the University Hospital clinical director before collecting the data.

## Result

A total of 250 ear discharge samples were analyzed at the University Hospital Microbiology Laboratory unit during the study period but only 228 (91.2%) of them had complete information for this analysis. Majority of the study participants were males (66.2% vs 33.8%). The mean age of the study participants was 18 (18 ±16) years with the minimum and maximum age of 10 months and 84 years old respectively. Majority of the study participants 58 (25.4%) were under five age groups.

The overall prevalence of bacterial isolates was 204 (89.5%). From the total bacterial isolates, 115 (56.4%) were gram negative bacteria. Of individuals who had bacterial isolates, 185 (90.7%) had single bacterial infection while 19 (9.3%) had mixed bacterial infections.

In this study, the predominant bacterial isolates were *proteus species* 56 (27.5%) followed by *S. aureus* 54 (26.5%). Majority (51 (25.0%)) of the bacterial isolates were found in under five age groups (P = 0.057). Males were more affected than females with significant difference (63.7 vs 36.3%) (P = 0.017) (Table 1).

**Table 1:** The distribution of bacterial isolates from ear discharge in different sex and age categories of study participants at Gondar University Hospital, Northwest Ethiopia (2009-2012)

**Age and sex**	**Bacterial isolates No(%)**									**P-value**
	***S. aureus***	**NLF GNR**	***E.coli***	***Pseud. spps***	**CNS*taph spps***	***Strep. spps***	***Prot. spps***	***Kleb. spps***	**Total**	
**Age in years**										
0–5	12 (23.5)	5 (9.8)	5 (9.8)	4 (7.8)	5 (9.8)	3 (5.9)	16 (31.4)	1 (2.0)	51 (25.0)	0.057
6–10	9 (29.0)	3 (9.7)	2 (6.5)	2 (6.5)	5 (16.1)	2 (6.5)	7 (22.6)	1 (3.2)	31 (15.2)	
11–15	5 (29.4)	0	0	1 (5.9)	1 (5.9)	2 (11.8)	8 (47.1)	0	17 (8.3)	
16–20	3 (13.6)	2 (9.1)	3 (13.6)	2 (9.1)	0	0	11 (50.0)	0	21 (10.3)	
21–30	14 (31.1)	3 (6.7)	0	3 (6.7)	6 (13.3)	3 (6.7)	8 (17.8)	8 (17.8)	45 (22.1)	
31–40	8 (38.1)	1 (4.8)	3 (14.3)	4 (19.0)	2 (9.5)	0	1 (4.8)	2 (9.5)	21 (10.3)	
≥41	3 (16.7)	1 (5.6)	1 (5.6)	2 (11.1)	4 (22.2)	2 (11.1)	5 (27.8)	0	18 (8.8)	
Total	54 (26.5)	15 (7.4)	14 (6.9)	18 (8.8)	23 (11.3)	12 (5.9)	56 (27.5)	12 (5.9)	204 (89.5)	
**Sex**										
Male	32 (24.6)	6 (4.6)	8 (6.2)	12 (9.2)	16 (12.3)	7 (5.4)	44 (33.8)	5 (3.8)	130 (63.7)	0.017
Female	22 (29.7)	9 (12.2)	6 (8.1)	6 (8.1)	7 (9.5)	5 (6.8)	12 (16.2)	7 (9.5)	74 (36.3)	

*S. aureusStaphylococcus aureus*, *NLF GNR* Non Lactose Fermenter Gram Negative rods, *Pseud. sppsPseudomonas species*, CN S*taph spps* Coagulase Negative *Staphylococcus species*, *Strep. spps Streptococcus species*, *Prot. spps Proteus species*, *Kleb. Spps Klebsella species.*

From 2,248 antibiotics which have been tested against the bacterial isolates, 871 (38.9%) had resistant pattern. Of these, 71.4% of E.coli was resistant for both ampicillin and amoxicillin, 75% of streptococcus species were resistant for tetracycline, 77.8% of the pseudomonas species were resistant for ampicillin and tetracycline, and 83.3% of the Klebsella species were resistant to ampicillin (Table 2). From the total bacterial isolates, 192 (94.1%) had multiple antibiotic resistant pattern (resistant to two or more antibiotics) and 10 (4.9%) of the isolates were resistant for at least one antibiotic. Only 2 (1.0%) bacterial isolates were susceptible to all antibiotics.

**Table 2:** Antimicrobial resistance pattern of bacterial isolates from ear discharge samples of study participants at Gondar University Hospital, Northwest Ethiopia (2009-2012)

**Bacterial isolates**	**Total NO**	**Resistance pattern of antimicrobial agents (R %)**										
		**AMP**	**AMX**	**CRO**	**CAF**	**CIP**	**ERY**	**CN**	**NOR**	**PG**	**SXT**	**TTC**
*S. aureus*	54	26 (48.1)	34 (63.0)	13 (24.1)	14 (25.9)	10 (18.2)	17 (31.5)	16 (29.6)	16 (29.6)	27 (50.0)	22 (40.7)	25 (46.3)
NLF GNR	15	9 (60.0)	10 (66.7)	4 (26.7)	11 (73.3)	4 (26.7)	6 (40.0)	5 (33.3)	2 (13.3)	6 (40.0)	9 (60.0)	10 (66.7)
*E.coli*	14	10 (71.4)	10 (71.4)	12 (50.0)	1 (7.1)	2 (14.3)	6 (42.9)	2 (14.3)	1 (7.1)	4 (28.6)	9 (64.3)	8 (57.1)
*Pseud. spps*	18	14 (77.8)	13 (72.2)	6 (33.3)	14 (77.8)	3 (16.7)	5 (27.8)	6 (33.3)	3 (16.7)	6 (33.3)	12 (66.7)	14 (77.8)
CN *staph spps*	23	9 (39.1)	3 (13.0)	5 (21.7)	7 (30.4)	5 (21.7)	5 (21.7)	5 (21.7)	6 (26.1)	6 (26.1)	11 (47.8)	11 (47.8)
*Strep.spps*	12	6 (50.0)	4 (33.3)	1 (8.3)	2 (16.7)	3 (25.0)	3 (25.0)	4 (33.3)	4 (33.3)	5 (41.7)	6 (50.0)	9 (75.0)
*Prot. spps*	56	31 (55.4)	24 (42.9)	17 (30.4)	32 (57.1)	10 (17.9)	13 (23.2)	12 (21.4)	12 (21.4)	19 (33.9)	23 (41.1)	44 (78.6)
*Kleb. spps*	12	10 (83.3)	8 (66.7)	7 (58.3)	5 (41.7)	4 (33.3)	4 (33.3)	4 (33.3)	3 (25.0)	7 (58.3)	6 (50.0)	5 (41.7)

*AMP* Ampicillin, *AMX* Amoxacillin, *CRO* Ceftriaxone, *CAF* Chloramphenicol, *CIP* Ciprofloxacin, *ERY* Erythromycin, *CN* Gentamycin, *NOR* Norfluxaciline, *PG* Penicillin G, *SXT* Co-trimoxazole, *TTC* Tetracycline.

## Discussion

Ear discharge is one of the most frequently ordered samples for microbiological analysis in the study area. This indicates that ear infection is a common problem in the given area. In this study, 89.5% cases of ear discharges were found to be positive for bacteria, which is in agreement with other studies in Ethiopia [9] and Nigeria [14]. Majority of the ear infection (56.4%) in the present study were caused by gram negative bacteria which is similar to previous studies that have been conducted in Ethiopia [9,15] and Nigeria [16]. In the present study, majority of the patients 185 (90.7%) had single bacterial infections which is similar to the other studies in Ethiopia [9] and Nigeria [14].

According to this study, majority of the bacterial isolates were found in under five years old children. A similar finding was also documented in previous studies [9,16,17]. This indicates that under five children were more affected by ear infections. This may be due to different factors such as anatomy of Eustachian tubes, the nutritional status of the children and other health problems like upper respiratory tract infections which are common in children [18].

There was significant difference on the prevalence of ear infections in genders. Males were more affected by ear infections than females (63.7 vs 36.3%) (P = 0.017). A similar finding was also reported by Egbe *et al*[19] but according to Hassan *et al* report [20], females were more affected by ear infections. This may be due to the difference between ear cleaning habit of the males and females. In some tradition, females use cotton swabs to clean their ear and this may contribute for the introduction of microorganisms from the external surface to the middle ear. However in some other studies [14,21], there is no difference on the prevalence of ear infections between males and females.

In this study, the predominant bacterial isolates were proteus species 56 (27.5%) followed by s. aureus 54 (26.5%) which is similar to previous study in Ethiopia [9]. However, in other studies [14,22], the predominant isolates were *Pseudomonas aeruginosa* and *s. aureus*. This may be due to the difference in climate and geographical variations in different countries. The other organisms which have been isolated in the present study in descending order were coagulase negative *staphylococcus species, pseudomonas species, Non lactose fermenter gram negative rods, E.coli, streptococcus species and Klebsella species.*

In the present study, different bacterial species had high level of resistance pattern to different antibiotics. For example, 71.4% of E.coli was resistant for both ampicillin and amoxicillin, 75% of streptococcus species were resistant for tetracycline, 77.8% of the pseudomonas species were resistant for ampicillin and tetracycline, and 83.3% of the Klebsella species were resistant to ampicillin. Similar finding were also reported in other studies [9,23-25]. Prescription of antibiotics without laboratory guidance and over sales of antibiotics without proper drug prescription may be some of the different factors that can contribute for this high level drug resistant pattern. Therefore, drug prescription for patients should be laboratory evidence based.

## Conclusion

In conclusion, the overall prevalence of bacterial isolates was high and majority of the isolates were gram negative bacteria. The predominant isolates were *Proteus species* and *S.aureus.* The bacteria which have been isolated from otitis media have shown high level of antibiotics resistance in the study area. Majority of the bacterial isolates had multiple antibiotic resistant patterns. Hence antibiotics susceptibility test is mandatory before prescribing any antibiotics.

### Limitation of the study

Due to the nature of the study, ear diagnosis is not clearly indicated and it is difficult to show whether the ear infection is acute otitis media with perforation, chronic suppurative otitis media, or otitis external. We are also unable correlate the bacterial findings with the severity of the infection. Some of the bacterial isolates were reported as non-lactose fermenting Gram negative rods and CN Staphylococci which are not specific. The isolated bacterial species were tested only for few antibiotics. In addition, there was no data about anaerobic bacteria and other fungal ear infections.

## Competing interest

The authors declared that no competing interest with respect to the authorship and/or publication of this research paper.

## Authors’ contributions

DM: participated in conception and design of the study, data collection and analysis, interpretation of the findings. YW: Participated in the design of the study, analysis and interpretations of the findings, drafting the manuscript and write up. GF: Participated in conception and design of the study, data analysis and interpretations of the findings. FM: Participated in conception and design of the study, data analysis and interpretations of the findings. TN: Participated in conception and design of the study and data collection. All authors reviewed and approved the final manuscript.

## Pre-publication history

The pre-publication history for this paper can be accessed here:

http://www.biomedcentral.com/1472-6815/13/10/prepub
